# Health impact of the first and second wave of COVID-19 and related restrictive measures among nursing home residents: a scoping review

**DOI:** 10.1186/s12913-022-08186-w

**Published:** 2022-07-15

**Authors:** Marjolein E. A. Verbiest, Annerieke Stoop, Aukelien Scheffelaar, Meriam M. Janssen, Leonieke C. van Boekel, Katrien G. Luijkx

**Affiliations:** Academic Collaborative Centre Older Adults, Tranzo Scientific Centre for Care and Wellbeing, Tilburg School of Social and Behavioral Sciences, Tilburg University, PO Box 90153, 5000 LE Tilburg, the Netherlands

**Keywords:** Coronavirus, SARS-CoV-2, Long-term care, Dementia, Person-centered care

## Abstract

**Background and objectives:**

COVID-19 disproportionally affects older adults living in nursing homes. The purpose of this review was to explore and map the scientific literature on the health impact of COVID-19 and related restrictive measures during the first and second wave among nursing home residents. A specific focus was placed on health data collected among nursing home residents themselves.

**Research design and methods:**

In this study, best practices for scoping reviews were followed. Five databases were systematically searched for peer-reviewed empirical studies published up until December 2020 in which data were collected among nursing home residents. Articles were categorized according to the type of health impact (physical, social and/or psychological) and study focus (impact of COVID-19 virus or related restrictive measures). Findings were presented using a narrative style.

**Results:**

Of 60 included studies, 57 examined the physical impact of COVID-19. All of these focused on the direct impact of the COVID-19 virus. These studies often used an observational design and quantitative data collection methods, such as swab testing or reviewing health records. Only three studies examined the psychological impact of COVID-19 of which one study focused on the impact of COVID-19-related restrictive measures. Findings were contradictory; both decreased and improved psychological wellbeing was found during the pandemic compared with before. No studies were found that examined the impact on social wellbeing and one study examined other health-related outcomes, including preference changes of nursing home residents in Advanced Care planning following the pandemic.

**Discussion and implications:**

Studies into the impact of the first and second wave of the COVID-19 pandemic among nursing home residents predominantly focused on the physical impact. Future studies into the psychological and social impact that collect data among residents themselves will provide more insight into their perspectives, such as lived experiences, wishes, needs and possibilities during later phases of the pandemic. These insights can inform policy makers and healthcare professionals in providing person-centered care during the remaining COVID-19 pandemic and in future crisis periods.

## Background and objectives

The new coronavirus - also known as Severe Acute Respiratory Syndrome coronavirus 2 (SARS-CoV-2) or COVID-19 (coronavirus disease 2019) - disproportionally affects older adults [1, 2]. In particular, adults aged 65 years and over who suffer from underlying chronic comorbidities, have functional difficulties in activities of daily living or experience cognitive decline are at risk of developing severe illness from a COVID-19 infection [2, 3]. Also, research has shown that people with dementia have a twofold increased risk of contracting COVID-19 compared to those without dementia. This can be explained by memory impairment that may interfere with adhering to preventive measures, such as social distancing, and frequent co-morbidities [4].

Living in a nursing home has also been found as an additional risk factor for infection and mortality due to a COVID-19 infection compared to living independently at home [3, 5]. A nursing home can be defined as “a facility with a domestic-styled environment that provides 24-hour functional support and care for persons who require assistance with activities of daily living and who often have complex health needs and increased vulnerability” [6]. Since crowding increases the risk for contamination, transmission of the virus is especially high in settings such as nursing homes [1, 7]. COVID-19-related deaths among residents of long-term care facilities, including nursing homes, represent 30–60% of all COVID-19 deaths in European countries [8]. The number of COVID-19-related deaths in nursing homes range from 19% in England and Wales (April/May 2020) up to 78% in Canada (August 2020) [2].

As a first response, nursing homes in many countries implemented restrictive measures during the first months of the COVID-19 pandemic in order to prevent an outbreak, such as strict no-visitor policies and placing infected residents into quarantine. Various studies show that such measures had a substantial impact on the health of residents, such as increased feelings of loneliness, psychological stress, apathy and depressive symptoms and may have outweighed the potential benefits of preventing further infections [9–12]. In a commentary, Mo & Chi conclude that loneliness and anxiety were the main psychological challenges faced by nursing home residents during the pandemic [13].

After a period of complete lockdown minor adjustments were made to these restrictions, such as allowing one visitor per resident per day. In-depth monitoring of nursing homes in the Netherlands in the first weeks after reopening the doors showed that allowing visitors again and restoring personal contact had a positive effect on the well-being of residents [14, 15]. By mid-2021, the majority of nursing home residents and staff in the Netherlands have been vaccinated [16]. Studies have shown an association between vaccination coverage rate in people over 65 and a reduced spread and a less severe clinical expression of COVID-19 [17]. Nevertheless, nursing homes are sometimes reluctant to further ease restrictions or, according to governmental policies, this process is moving gradually to “a new normal” in which social distancing and hygiene measures are still in place [16]. Consequently, to date the COVID-19 pandemic continues to impact the lives of nursing home residents.

Since the outbreak of the pandemic, numerous studies have examined the impact of COVID-19 and related restrictive measures on health. In line with the biopsychosocial model, which states that illness and health are the result of an interaction between biological, psychological, and social factors [18], these studies have not only focused on the biological or physical impact (e.g. infection rates, mortality or symptoms), but also on the psychological impact (e.g. mood or behavior) and social impact (e.g. loneliness). With regard to the impact of COVID-19 on nursing home residents, Thomson et al. have previously synthesized research that focused on the physical health impact of the COVID-19 virus [2]. The purpose of this review is therefore not to replicate this study and provide insight into, for example, specific infection and mortality rates. However, we aim to describe the published research on the overall health impact of the first and second wave of COVID-19 and related restrictive measures on nursing home residents, including the physical, psychological and social health impact. In order to capture the health impact from the perspectives of nursing home residents themselves, we only including studies that collected data among residents themselves, both objective data (e.g. data from health records or swab tests) as well as subjective data (e.g. data based on interviews or questionnaires).

## Research design and methods

A scoping review was conducted in order to explore and map the scientific literature on the impact of COVID-19 and related restrictive measures on nursing home residents. As many countries have seen a two-wave pattern in reported cases of COVID-19 during 2020, with a first wave during spring followed by a current second wave in late summer and autumn, we focused on studies published during the first and second waves of the pandemic (up until December 2020) [19]. This scoping review was reported following PRISMA-ScR reporting guidelines [20].

The methodological steps outlined in the framework of Arksey and O’Malley for conducting scoping reviews were followed to undertake this review [21]. This framework includes the following steps: (i) identifying the research question, (ii) identifying relevant studies, (iii) selecting appropriate studies, (iv) charting the data and (v) collating, summarising and reporting the results.

### Identifying the research question

The following research questions were identified:

1. What types of health impact of the first and second wave of COVID-19 and related restrictive measures on nursing home residents have been studied?
2. Which knowledge gaps can be identified regarding the impact of COVID-19 and related measures on the health of nursing home residents?

### Selecting appropriate studies

Five scientific databases were systematically searched including PsychInfo, PubMed, CINAHL, Science Direct and Google Scholar using a search string of three categories of key words related to ‘COVID-19’, ‘nursing home’, and ‘older adults’ (Appendix 1 and 2). The databases were searched for scientific English language papers published between the start of the COVID-19 pandemic and December 2020. Studies were eligible for inclusion if they met de following criteria:

1. Studies that examined the impact of COVID-19 on nursing home residents. If mixed target groups were included or compared (e.g. nursing home residents versus community dwelling older people or the general population), we only focused on study findings related to nursing home residents;
2. Studies that examined the health impact of COVID-19 on nursing home residents, including physical, psychological and social health;
3. Studies that examined either a direct (e.g. infection or mortality) or indirect (e.g. impact of COVID-9 related measures of restrictions) impact of COVID-19;
4. Data were collected among residents themselves (e.g. data from health records, such as symptoms, blood tests and other diagnostics), observations, questionnaires, interviews);
5. Original empirical, peer-reviewed studies;
6. Qualitative, quantitative and mixed methods studies.

We excluded studies that collected data among proxies (e.g. health professionals or family members) in order to ensure we captured the health impact from the perspectives of nursing home residents themselves. Also, we excluded studies that focused exclusively on community-dwelling older adults or were not written in English.

Selected articles from the different databases were imported into Endnote. After duplicates were removed, one of the authors (MV, AS1, AS2, LvB or MJ) screened all unique titles and abstracts. In any case of doubt, the other authors were consulted to reach consensus. Next, the remaining full text articles were screened by one of the authors (MV, AS1, AS2, MJ) for inclusion following a comparable approach. During the screening process, several joint meetings took place to ensure that all reviewers used the screening criteria in the same way.

### Charting the data

A data-extraction sheet was developed and for each included article, one of the authors (MV, AS1, AS2, MJ) extracted the following data: publication year and month, country, study design, period of data collection or duration of the study, method of data collection, sample size of included nursing homes and residents, and basic demographics of residents (gender and age). Articles were categorized according to the type of impact of COVID-19 the study focused on. Following the biopsychosocial model of health [18], studies were classified into either studying the physical health impact (e.g. infection rates, mortality or symptoms), the psychological health impact (e.g. mood, behavior, quality of life or overall wellbeing) and/or the social impact (e.g. loneliness). Also, included articles were assigned to either studying the direct health impact of the COVID-19 virus (e.g. following an COVID-19 infection) or the health impact of COVID-19-related restrictive measures, such as the lockdown, visitor restrictions or social distancing. Finally, the type of health outcome that was studied was extracted from each article. Examples of such health outcomes are the number of COVID-19 cases or infection rates, mortality rates, loneliness or depression.

### Collating, summarising and reporting the results

Findings are presented using a narrative style. A descriptive summary of basic study characteristics (e.g. country, study design, sample size, data collection method, outcome) is provided and presented using visual representation including a table and bar chart. Results from studies solely focusing on the physical impact of COVID-19 are not reported, as this was not the primary aim of this review and was recently reported by others [2]. In order to get insight into the health impact of COVID-19 from the perspectives of nursing home residents, however, results from studies on the psychological and social health impact more described in-depth.

## Results

### Types of studies

**Fig. 1:**
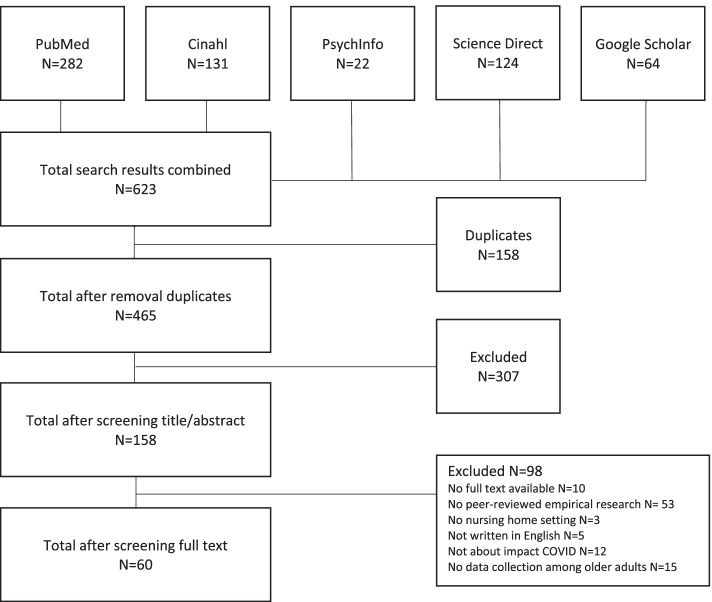
Flowchart of included studies

Figure 1 summarizes the study selection results. The initial search resulted in 465 unique articles. After titles and abstracts were screened, 158 full text articles were left and screened for further inclusion. This resulted in a total of 60 included articles. Most excluded articles were not peer reviewed and/or not based on empirical research (*n* = 53).

**Fig. 2:**
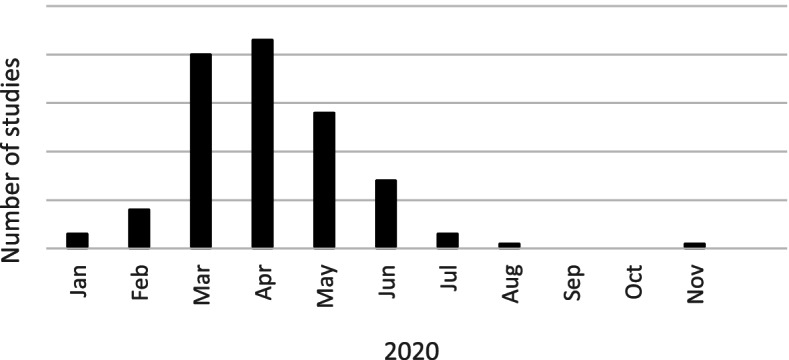
Data collection period of included studies

Table 1 provides an overview of the characteristics of the included studies. The studies were performed in 12 different countries, of which the majority in Europe (*N* = 29, 48%) and the USA (*n* = 25, 42%). Only six studies were performed elsewhere, namely in Canada [7, 22–25] and South Korea [26]. The number of nursing homes per study varied significantly across studies, ranging from one [27–42] to 12,576 nursing homes [43]. The number of residents per study ranged from four [27] to 696,310 [44]. Data collection periods spanned from January to November 2020, with the majority of studies conducted between March and May 2020 (Fig. 2).

**Table 1:** Characteristics of included studies on the impact of COVID-19 and related restrictive measures on nursing home residents

Authors, publication year	Country	Study design	Study period	NH (n)	Residents (n)	Data collection method^a^	Impact type	Health outcome^b^
Abrams et al. 2020 [45]	USA	Cross-sectional study	March-April 2020	9,395		Database	Physical	Infection
Beilenson, 2020 [30]	USA	Case series	April 2020	1	39	Swabs	Physical	Infection
Belmin et al., 2020 [44]	France	Retrospective cohort study	March-May 2020	17 (self-confinement) 9,513 (national survey)	1,250 (self-confinement) 695,060 (national survey)	Swabs	Physical	Infection, mortality
Bernabeu-Wittel et al., 2020 [46]	Spain	Non-randomised controlled trial	April 2020	4	457	Swabs, serological blood tests	Physical	Infection, symptoms, complications, survival
Bielza et al., 2020 [47]	Spain	Retrospective cohort study	March-June 2020	55	630	Swabs	Physical	Infection, mortality, mortality risk, symptoms
Blain et al., 2020 [31]	France	Prospective cohort study	March 2020	1	79	Swabs	Physical	Infection, symptoms
Blain et al., 2020 [32]	France	Prospective cohort study	March 2020	1	79	Swabs	Physical	Symptoms
Braun et al., 2020 [48]	USA	Cross-sectional study	May-July 2020	11,470		Databases	Physical, other	Infection, mortality, staff shortages
Brouns et al., 2020 [49]	The Netherlands	Retrospective case series	March-May 2020	14	101	Swabs, electronic health records	Physical	Mortality
Brown et al., 2020 [7]	Canada	Retrospective cohort study	March-May 2020	618	78,607	Databases, Swabs	Physical	Infection, mortality
Buntinx et al., 2020 [36]	Belgium	Case series	March-April 2020	1	119	Swabs, serological blood tests	Physical	Infection
Burton et al., 2020 [50]	UK	Prospective cohort study	March-August 2020	189	5,227	Electronic health records	Physical	Infection, mortality
Carta et al., 2020 [33]	Italy	Prospective cohort study	March-April 2020	1	65	Swabs, serological blood testing	Physical	Infection, mortality
Collison et al., 2020 [37]	USA	Cross-sectional study	April 2020	1	120	Swabs	Physical	Infection
Dini et al., 2020 [51]	Italy	Cross-sectional study	April-May 2020	12	150	Swabs, lung ultrasound	Physical	Infection, symptoms
Dora et al., 2020 [40]	USA	Prospective cohort study	March-June 2020	1	150	Swabs, serological blood tests, provider notes	Physical	Infection, symptoms
Echeverria et al., 2020 [52]	Spain	Prospective cohort study	April 2020	169	10,347	Swabs, CovidApp	Physical	Infection, symptoms,, mortaility
Eckardt et al., 2020 [38]	USA	Cross-sectional study	April-May 2020	1	120	Swabs	Physical	Infection
El Haj et al., 2020 [53]	France	Cross-sectional study	-	-	58	Survey	Psychological	Mental health
Escobar et al., 2020 [34]	USA	Case series	March-April 2020	1	84	Swabs	Physical	Infection
Feaster & Goh, 2020 [54]	USA	Prevalence study	April 2020	9	582	Swabs, electronic health records, case reports, facility records	Physical	Infection
Fischer, 2020 [29]	USA	Observational case series	April-June 2020	1	38	Swabs, electronic medical records	Physical	Viral shedding, case duration, mortality, risk of clinical deterioration
Fisman et al., 2020 [25]	Canada	Cohort study	March-April 2020	627	79,498	Database	Physical	Infection, mortality
Girolamo et al., 2020 [55]	Italy	1. Prevalence study 2. Retrospective cohort study	February-April 2020	1. 3,276 2. 162	1. 80,131 2. 16,000	1. Databases 2. Database	Physical	1. Mortality 2. Observed/ expected mortality ratio
Graham et al., 2020 [56]	UK	Prevalence study	March-May 2020	4	394	Swabs, clinical and demographic data collection	Physical	Infection, symptoms, mortality
Graham et al., 2020 [57]	UK	Outbreak investigation	June 2020	4	173	Serological blood tests	Physical	Infection
He et al., 2020 [58]	USA	Cross-sectional study	June 2020	1223	n/a	Database	Physical	Infection, mortality
Heudorf et al., 2020 [59]	Germany	Case studies	March-April 2020	40	116	Database	Physical	Infection, mortality
Hollinghurst et al., 2020 [60]	UK	Cross-sectional cohort study	March-June 2020	544	12,568	Electronic health records, database	Physical	Mortality
Kennelly et al., 2020 [61]	UK	Cross-sectional study	February-May 2020	28	2,043	Survey	Physical	Outbreak (nursing home level), infection, mortality, case-fatality rate, recovery
Kittang et al., 2020 [62]	Norway	Retrospective observational study	March-April 2020	3	115	Swabs	Physical	Asymptomatic during diagnosis, symptoms, mortality, recovery, hospitalization
Ladhani et al., 2020 [63]	UK	Cohort study	March-April 2020	6	264	Swabs, staff recorded symptoms, care home data	Physical	Infection, hospitalization, mortality, CT values, recovery
Ladhani et al., 2020 [64]	UK	Prospective cohort study	November 2020	6	186	Swabs, serological blood test	Physical	Infection
Li et al., 2020 [43]	USA	Cross-sectional cohort study	May 2020	12,576	-	Databases, number of cases and deaths as publishes by New York Times	Physical	Infection, mortality
Li et al., 2020 [65]	USA	Cross-sectional cohort study	Up to April 2020	215	-	Databases, number of cases and deaths as publishes by New York Times	Physical	Infection, mortality
Lipsitz et al., 2020 [66]	USA	Longitudinal cohort study	May-July 2020	360	-	Nursing home audits, nursing facility reports	Physical	Infection, hospitalization, mortality
Louie et al., 2020 [67]	USA	Cross-sectional study	March-April 2020	4	-	Swabs	Physical	Infection, hospitalization, mortality
Ly et al., 2020 [68]	France	Cross-sectional study	March-June 2020	83	1,691	Electronic health records, interviews	Physical	Infection, mortality
Marossy et al., 2020 [69]	UK	Cross-sectional study	May 2020	37	1,034	Swabs	Physical	Infection, symptoms, CT values
Martinchek et al., 2020 [41]	USA	Retrospective chart review	March-May 2020	1	209	Electronic medical record, swabs	Physical	Weight loss
Mas Romero et al., 2020 [70]	Spain	Cross-sectional study	March-April 2020	6	1,260	Interviews, electronic medical records	Physical, other	Mortality
Matias-Guiu et al., 2020 [71]	Spain	Case series	May-June 2020	-	25	Electronic health records	Physical	Infection, mortality, symptoms, hospitalization
McArthur et al., 2020 [22]	Canada	Retrospective chart review	January 2017 - June 2020	7	765	Electronic health records	Psycho-logical, behavioural	Depression, delirium and behavioural problems
McConeghy et al., 2020 [72]	USA	Retrospective cohort study	February-June 2020	416	4,669	Electronic health records	Physical	Infection, temperature changes
McMichael et al., 2020 [73]	USA	1. Case series 2. Cross-sectional study	February-March 2020	1. 1 2. 100	101	1. Databases, swabs, diagnostic testing, interviews 2. Survey	Physical	1. Infection, mortality, chronic underlying conditions 2. Factors contributing to vulnerability of infections
Montoya et al., 2020 [74]	USA	Retrospective cohort study	March-April 2020	3	215	Electronic health records, swabs	Physical	infection, mortality, symptoms, co-morbidities, hospitalization
Österdahl et al., 2020 [28]	USA	Observational study	February-March 2020	1	21	Swabs	Physical	Infection, symptoms, mortality
Park et al., 2020 [26]	South Korea	Case series	March 2020	3	596	Swabs, databases	Physical	Infection, symptoms, mortality
Patel et al., 2020 [39]	USA	Prospective cohort study	March 2020	1	126	Swabs, interviews with residents, electronic health records	Physical	infection, symptoms, hospitalization, mortality
Rawle et al., 2020 [75]	UK	Retrospective cohort study	March-April 2020	-	64	Electronic health records	Physical	Symptoms, mortality
Rudolph et al., 2020 [76]	USA	Cohort study	March-May 2020	134	7,325	Electronic health records	Physical	Infection, symptoms, temperature
Rutten et al., 2020 [77]	The Netherlands	Prospective cohort study	March-May 2020	-	4,007	Swabs, electronic health records	Physical	Mortality, symptoms, risk factors for mortality
Sacco et al., 2020 [35]	France	Retrospective cohort study	March-April 2020	1	87	Electronic health records	Physical	Infection, mortality, attack rate, symptoms
Shi et al., 2020 [42]	USA	Retrospective cohort study	March-May 2020	1	389	Electronic health records, minimum data set	Physical	Mortality, infection, symptoms
Stall et al., 2020 [23]	Canada	Retrospective cohort study	March-May 2020	623	75,676	Electronic health records	Physical	Number of outbreaks, infection, mortality
Strang et al., 2020 [78]	Sweden	Observational study	March-April 2020	-	253	Electronic health records	Physical	Mortality, symptoms
Tang et al., 2020 [79]	USA	Retrospective cohort study	March-June 2020	15	1,970	Electronic health records	Physical	Infection, symptoms, mortality, hospitalization
Wang et al., 2020 [24]	Canada	Prospective cohort study	January-May 2020	-	28,316	Databases	Physical	Proportion of people tested, infection, mortality
Ward et al., 2020 [27]	USA	Case series	-	1	4	Swabs, electronic health records, interviews	Physical, psychological	Infection, mental health, confusion
Ye et al., 2020 [80]	USA	Retrospective chart review	April-May2020	15	963	Advanced Care Planning conversations	Other	Code status, goals of care

*NH *nursing home, *CT *cycle threshold

^a^Only data collected methods among residents are reported

^b^Only health outcomes among residents are reported

Most studies used an observational study design to examine the impact of COVID-19 on nursing home residents (*n* = 59, 98%), such as cross-sectional (n = 15, 25%), retrospective (*n* = 15, 25%) or prospective (*n* = 12, 20%) studies. Also, 7 case series were included [26, 27, 30, 34, 36, 59, 73]. Only one study used an experimental, non-randomised study design [46].

### Impact of COVID-19 and related restrictive measures

#### Physical impact

Of the 60 included studies, 57 (95%) focused on the physical impact of COVID-19 on nursing home residents. All of these focused on the direct health impact of the COVID-19 virus. The most common outcomes were infection (*N* = 46, 77%) and mortality (*N* = 39, 65%). Other outcomes were symptoms (*N* = 22, 37%) and hospitalization (*N* = 8, 13%). Less frequently, studies focused on cycle threshold values (i.e. an estimate of how much virus was likely in the sample of a PCR test) (*N* = 2) [63, 69], the number of outbreaks (*N* = 1) [23] or temperature (*N* = 2) [72, 76]. One study focused on the impact of COVID-19 on weight loss [41]. Data was mainly collected using swab testing (*n* = 32, 56%) or electronic health records (*n* = 21, 37%).

A variety of data collection methods were used in these studies. Most studies used quantitative methods (*n* = 52, 91%); over half of the studies used swab testing (*n* = 32, 56%), followed by a third of the studies that extracted physical data from electronic health records (*n* = 21, 37%). Other quantitative data collection methods included (linked) databases (*n* = 14, 25%), serological blood testing (*n* = 6, 11%) [33, 36, 40, 46, 57, 63] and surveys (*n* = 2, 4%) [61, 73]. Only a minority of these studies used qualitative data collection methods (*n* = 5, 9%); four studies used interviews with residents [39, 68, 70, 73] and one study collected data regarding symptoms through review of provider notes (i.e. notes of healthcare providers in electronic health records) [40]. Other qualitative and quantitative data collection methods used within these studies included for example lung ultrasound [51], a COVIDapp [52] and case reports [54].

Most studies that examined the physical impact of COVID-19 on nursing home residents used an observational study design, such as a prospective or retrospective cohort study designs (*n* = 26, 46%) or a cross-sectional study design (*n* = 14, 25%). For example, Rutten et al. performed a large (*N* = 4007) prospective cohort study among Dutch nursing home residents with clinically suspected COVID-19 who where tested with a reverse transcription-polymerase chain reaction swab test in order to describe the symptomology, mortality and risk factors for mortality [77]. In this study, data were collected using electronic health records between March and May 2020 and showed that COVID-19 was confirmed in 38% of residents. Positive tested residents had a three-fold increased risk of death compared to residents who were tested negative.

Several other studies used a case series design in which a COVID-19 outbreak in a particular nursing home was described (*n* = 7, 12%) [26, 27, 30, 34, 36, 59, 73]. For example, an outbreak in an assisted living facility with 39 residents in Sacramento, California was studied [30]. In this case series, 25 of 39 residents aged 72 to 99 were tested positive of which 11 required hospitalization. Two residents deceased due to COVID-19, age and underlying chronic conditions.

The only experimental study that was included was a non-randomised controlled study that examined the effectiveness of a coordinated, on-site medicalization programme in four Spanish nursing homes with the objective to improve survival, offering humanistic palliative care to residents in their natural environment, and reduce hospital referrals [46].

#### Psychological impact

Three studies (5%) that met our inclusion criteria focused on the psychological impact of COVID-19 on nursing home residents [22, 27, 53]. These studies were performed in France [53], Canada [22] and the USA [27] and varied with regard to sample size, study design and data collection methods. They also differed with regard to their specific study focus; one study focused on the psychological impact related to a COVID-19 infection [27] and two studies focused on the psychological impact of COVID-19-related restrictive measures, such as a lockdown or visitor restrictions [22, 53].

El Haj et al. [53] performed a cross-sectional study using survey data collected among residents with dementia (*N* = 58) regarding the impact of COVID-19-related measures (social distancing) on their psychological wellbeing. McArthur et al. [22] conducted a retrospective, observational study using data from routinely collected, quarterly assessments of psychological wellbeing and behavior of residents with and without dementia (*N* = 765), between January 2017 and June 2020. The assessment tool was administered by trained registered nurses through interaction with residents. Findings of both studies were contradictory; whereas El Haj et al. found *higher* depression and anxiety among residents during than before COVID-19, McArthur et al. found *no change* in the number of residents that experienced a delirium or behavioral problems and a *decrease* in the proportion of residents with indications of depression during than before lockdown. A third article by Ward et al. [27] describes a case series including four residents with dementia. Based on data from electronic health records, the study found that these residents developed an altered mental status, such as confusion, agitation, refusing care, disorientation and loss of appetite, just before a positive COVID-19 test. This may suggest that altered mental status is one of the first signs of a COVID-19 infection among residents with dementia.

#### Social impact

No studies have been published on the social impact of the first and second wave of COVID-19 and related restrictive measures on nursing home residents that collected among residents themselves.

#### Other outcomes

One study was included that examined the impact of COVID-19 among nursing home residents, other than the physical, psychological or social health impact. This retrospective chart review examined the impact of the COVID-19 pandemic on goals of care within Advanced Care Planning [80]. Based on electronic health records, the study found that proactive Advanced Care Planning conversations with residents (or their surrogate decision makers) between April and May 2020 increased ‘Do Not Hospitalize’ care preferences from less than a quarter to almost half among residents.

## Discussion and implications

This review aimed to scope the scientific literature published on the health impact of COVID-19 and related restrictive measures during the first and second wave of the pandemic among nursing home residents. To focus on the perspectives of residents - which provides insights into their lived experiences, wishes and needs during the COVID-19 pandemic - studies were only included if data were collected among nursing home residents. This included both objective data (e.g. data from health records or swab tests) as well as subjective data (e.g. data based on questionnaires). It was found that the direct impact of the COVID-19 virus on physical health among nursing home residents was studied extensively. In particular, a large number of studies - predominantly from Europe and the USA - examined the impact of COVID-19 on infection and mortality rates, symptoms and hospitalization. These numbers have been previously collated and show that the number of COVID-19-related deaths in care homes ranged from 18.6% in England and Wales (April/May 2020) to 78.2% in Canada (August/September 2020) out of deaths in total [2].

From the perspectives of nursing home residents themselves, we identified a major knowledge gap with regard to the psychological and social impact of the first and second wave of COVID-19 and related restrictive measures. Only three included articles published in that period studied the psychological impact using data collected from nursing home residents. These studies varied in sample size, study design, data collection methods and study focus, i.e. one study focused on the psychological health impact related to a COVID-19 infection and two studies focused on the impact of COVID-19-related restrictive measures. The findings were contradictory. At the one hand, residents with dementia reported higher depression and anxiety during than before the COVID-19 crisis [53] and an altered mental status was found as a symptom of a COVID-19 infection among persons with dementia [27]. At the other hand, it was found that the proportion of residents (with and without dementia) with indications of depression decreased during lockdown [22]. Based on the limited number of studies and the contrasting findings, we urge researchers to further study the psychological impact of later phases of the COVID-19 pandemic on nursing home residents as well as the long-term psychological impact. Regarding the social impact of COVID-19, no studies were found that collected data from nursing home residents and focused on the impact of COVID-19 on social wellbeing. Therefore, we also recommend further research into this area.

Whereas physical health outcomes can be studied using relatively straightforward procedures, studying social and psychological aspects of health among people with cognitive impairment can be much more challenging. Since we only included studies that collected data among nursing residents themselves, this may explain why we identified these knowledge gaps.

### Strenghts and limitations

This scoping review is the first that provides insight into empirical studies on the overall health impact of COVID-19 and related restrictive measures among nursing home residents published during the first and second wave of the pandemic in 2020. A broad search strategy was used to include studies examining both the physical impact as well as the psychological and social impact. However, several limitations of this review have to be mentioned.

First, we searched for literature published in a relatively short timeframe - between the beginning of the pandemic up until December 2020. It may be possible that we have missed studies that examined the impact of the first and second wave because of a delay between acceptance and actual publication dates. Also, because we focused on studies that collected data among residents themselves, which often involves time-consuming ethical approval procedures, such studies may not yet have taken place or were not yet published within this timeframe. This may also explain why the publication date of the included studies drops off as 2020 progresses. Future updates of this review will most likely include more relevant studies that were published after December 2020 and will provide further insight into the impact of later phases of the COVID-19 pandemic on residents’ health.

Second, we only included empirical and peer-reviewed studies to ensure a high-quality standard. Because grey literature was not included, we may have missed relevant reports, papers or other non-academic material published. For example, we excluded a commentary of Mo & Shi from China providing an overview of existing literature on the psychological consequences caused by COVID-19 among residents of nursing homes [13]. To maintain the high-quality standard we, however, recommend future updates to include only empirical and peer-reviewed studies.

Third, we aimed to perform a rapid literature search given the fast developments regarding the COVID-19 pandemic. As such, the search was not performed by a librarian and only search terms were used, i.e. no MeSH/indexed terms. Also, for similar pragmatic reasons, screening and data extraction was performed by only one of the authors, which may be considered as a limitation as independency cannot be guaranteed. However, any doubts regarding inclusion of articles and data extraction were discussed jointly with other authors. Nevertheless, we recommend that future iterations of this review should include more thorough search strategies and ideally involve two authors in the screening and data extraction procedures.

Lastly, the majority of included studies collected data during the first months of the pandemic – March to May 2020. Therefore, the results specifically relate to the impact of the first wave of the COVID-19 pandemic in which whole societies were in lockdown and nursing homes had to deal with frequent outbreaks, shortage in equipment, limited knowledge about consequences and treatment, and strict restrictive measures. It is likely that studies published in later phases of the pandemic provide additional (and potentially other) findings on the health impact among nursing home residents. Future updates of this scoping review are therefore recommended in order to include new studies and to compare the current findings to the health impact of later phases of the COVID-19 pandemic, in which related restrictive measures were eased and to the long-term health impact of COVID-19 and related measures on nursing home residents.

### Implications

We found that studies into the perspectives of nursing home residents regarding the psychological and social impact of COVID-19 are scarce. Data collection among nursing home residents with a form of cognitive impairment or dementia – which make up for the majority of nursing home residents – can provide important insights into their lived experiences, wishes, needs and possibilities. This aligns with the current paradigm shift in nursing home care, in which care is shifting from a biomedical care model towards a person-centered care model [81, 82]. Person-centered care recognizes each person’s unique identity, preferences and needs and enables older adults to continue their preferred lives whilst living in a nursing home, also during crisis periods such as the COVID-19 pandemic. Being sensitive to individual needs and wishes is an essential prerequisite for person-centered care. Although it can be challenging, both ethically and practically, previous research has shown that data collection among older adults with complex care needs, such as dementia, is possible and provides important insights into their perspectives [83–86]. Examples of such data collection methods are interviews, observation methods (e.g. dementia care mapping, shadowing, video observations) and diary interviewing. Nevertheless, the perspectives of older adults have been scarcely studied during disasters or crises to date [87]. Although studies using proxies provide important insights, the perspectives of nursing home residents and those of proxies can differ [88]. This difference may affect the resident’s experience of feeling heard, seen, and respected as a unique individual in the care and support he or she receives. Future research into the perspectives of older adults living in nursing homes are therefore needed to fill the knowledge gap and further guide healthcare professionals in providing person-centered care during the remaining COVID-19 pandemic, but also in future crisis periods.

By focusing exclusively on data collected among residents, however, we may have missed important studies that used a proxy to collect data. During the screening process, we indeed excluded a few studies that collected data on the impact of COVID-19 on the psychological and/or social wellbeing of nursing home residents among proxies. For example, Leontjevas et al. examined the impact of COVID-19 on challenging behaviors and psychological wellbeing of Dutch nursing home residents [89]. Data were collected between April and June 2020 using an online survey among nursing home psychologists, elderly care physicians and nurse practitioners. Both increased and decreased challenging behaviors were found, including, amongst others, increased depression and loneliness as well as elevated mood. Although findings of this study are considered relevant, we recommend future studies that involve residents themselves in the data collection to capture their own perspectives.

Because this scoping review focused on literature published during the first and second wave of the COVID-19 pandemic in 2020, we also recommend future research that shed light on the overall long-term impact of the pandemic, as well as on the impact of easing restrictions. For example, Koopmans et al., studied the impact of reopening the doors of Dutch nursing homes during the COVID-19 crisis and allowing visitors again after a 2-month lockdown period. An online survey, regarding changes in resident wellbeing that were observed after the lockdown, was completed by nurses, psychologists and physicians of 26 nursing homes. Although overall visitors and staff noticed that residents seemed to enjoy the visits, became overall more active and returned to their units in good spirit after a visit, some residents experienced sadness because physical contact was prohibited. In some residents with dementia, visits seemed to result in confusion, sadness or restlessness. Consequently, staff advised against visiting these residents anymore under the circumstances of that time [14].

## Conclusions

Studies on the impact of COVID-19 and related restrictive measures, such as visitor restrictions and social distancing, among nursing home residents published in the first and second wave of the pandemic in 2020 focused predominantly on the physical health impact of the virus, including infection and mortality rates. Consequently, a knowledge gap exists regarding the psychological and social health impact, in particular from the perspectives of nursing home residents themselves. Therefore, we recommend further empirical studies into the impact of later phases of the COVID-19 pandemic and the long-term impact on social and psychological wellbeing of nursing home residents, with a specific focus on their own perspectives. Giving nursing home residents a voice contributes to improving person-centered care and support that is tailored to their needs and preferences during the remaining COVID-19 pandemic and in future crises [90].

## Data Availability

Data available on request from the authors.

## Appendix 1

Table 2

**Table 2:** Databases

Database	Focus	Filters
PubMed	Biomedical literature	English, year 2015-present, title/abstract
PsycINFO	Psychological literature	English, year 2015-present
CINAHL	Nursing literature	English, year 2015-present
Science Direct	Biomedical literature	English, year 2015-present, title/abstract
Google Scholar	Scholarly literature	English, year 2015-present, title

## Appendix 2

Table 3

**Table 3:** Search terms

**COVID-19**
“covid” OR “covid 19” OR “cov 2” OR “corona” OR “coronavirus” OR “coronavirus” OR “covid-19” OR “sars cov” OR “sars cov 2” OR “sars-cov” OR “sars-cov 2” OR “sars cov-2”
**Nursing homes**
“Residential facilities” OR “long term care” OR “residential care” OR “nursing home” OR “homes for the aged” OR “assisted living facilities” OR “care home” OR “institutional care”
**Older adults**
“older adults” OR “residents” OR “older patients” OR “inpatients” OR “geriatric patients” OR “elderly”

## Abbreviations

COVID-19: Coronavirus disease 2019
MeSH: Medical Subject Headings
PRISMA-ScR: Preferred reporting items for systematic reviews and meta-analyses extension for scoping reviews
SARS-CoV-2: Severe acute respiratory syndrome coronavirus 2
USA: United States of America
